# Widespread Distribution of *chs-1* Mutations Associated with Resistance to Diflubenzuron Larvicide in *Culex pipiens* Across Italy, Reaching Virtual Fixation in the Venetian Lagoon

**DOI:** 10.3390/insects16020204

**Published:** 2025-02-12

**Authors:** Martina Micocci, Verena Pichler, Paola Serini, Carola Giammarioli, Chiara Malandruccolo, Chiara Virgillito, Marco Ballardini, Riccardo Paolo Lia, Daniele Arnoldi, Stefano Vettore, Davide Bonetto, Simone Martini, Andrea Drago, Alessandra della Torre, Beniamino Caputo

**Affiliations:** 1 Department of Public Health and Infectious Diseases, Sapienza University of Rome, 00185 Rome, Italy; martina.micocci@uniroma1.it (M.M.); verena.pichler@uniroma1.it (V.P.); paola.serini@uniroma1.it (P.S.); chiara.virgillito@uniroma1.it (C.V.); 2 Istituto Zooprofilattico Sperimentale del Piemonte, Liguria e Valle d’Aosta, 10154 Torino, Italy; marcoballardini@yahoo.it; 3 Department of Veterinary Medicine, University of Bari “Aldo Moro”, 70121 Valenzano, Italy; riccardopaolo.lia@uniba.it; 4 Research and Innovation Centre, Fondazione Edmund Mach, San Michele all’Adige, 38098 Trento, Italy; daniele.arnoldi@fmach.it; 5 Entostudio Srl, Ponte San Nicolò, 35020 Padova, Italy; vettore@entostudio.com (S.V.); bonetto@entostudio.com (D.B.); martini@entostudio.com (S.M.); drago@entostudio.com (A.D.)

**Keywords:** mosquito, larval control, insecticide resistance, *chs-1* mutation genotyping, diflubenzuron, *Culex pipiens*

## Abstract

Control interventions against mosquito larvae are the primary measure to reduce the adult abundance and risk of arbovirus outbreaks in Europe. One of the most commonly used larvicides in Italy is diflubenzuron (DFB), which targets chitin synthase 1 (*chs-1*), interrupting the normal development of larvae into adults. Recent studies identified high levels of DFB resistance in *Culex pipiens* populations from Emilia-Romagna (Italy) associated with I1043L/M/F mutations at position 1043 of the *chs-1* gene. The aim of the present study was to assess the circulation of 1043 resistance alleles in *Cx. pipiens* populations across Italy, outside Emilia-Romagna, with a focus on the Veneto region. Overall, 1032 specimens were genotyped. The 1043L allele was found in all examined Italian regions (Trentino-Alto Adige 19–36%; Veneto 0–91%; Piemonte 11%; Liguria 28%; Lazio 0–8%; Puglia 5%). The highest frequencies (up to >90%) were observed in the Venetian lagoon, where 1043M was also detected (6–11%). Overall, the relatively low frequencies of 1043 mutations despite extensive and longstanding use of DFB in Italy suggest a high fitness cost worthy of further investigations, while their extremely high frequencies in coastal touristic sites point to these sites as the most relevant for resistance monitoring and larvicide rotation.

## Simple Summary

Mosquitoes are a major problem worldwide not only for the nuisance they cause but also as they transmit numerous pathogens. In European urban areas, rain catch basins represent one of the preferred sites for the larval development of the common mosquito—Culex pipiens—which is responsible for the transmission of West Nile virus, causing hundreds of human infections and a few deaths every year. Public administrations often treat these habitats with insecticides to reduce the nuisance and risk of disease transmission. One of the most commonly used products is diflubenzuron, which prevents the development of larvae into adults. The effectiveness of diflubenzuron treatments is, however, threatened by the development of mosquito populations resistant to the product. This resistance can be detected by signatures in the mosquito DNA. We investigated the presence of these DNA signatures in Culex pipiens populations across Italy, with a focus on the Veneto region. The results show that they are widespread, but most frequent in mosquito populations collected in coastal sites, such as sites in the Venetian lagoon. These results point to the need to frequently change the insecticide product at these sites in order to prevent development of highly resistant mosquito populations and maintain a high effectiveness of the implemented treatments.

## 1. Introduction

The indigenous *Culex pipiens* is one of the most abundant mosquito species in Europe and the principal vector of West Nile virus (WNV) in southern and southeastern regions of the continent [1]. In Italy, *Cx. pipiens* plays a primary role as vector of WNV [2,3], with the highest epidemiological impact [4]. West Nile virus has become endemic in several countries in Europe [5], with the largest European WNV epidemic in the summer of 2018, with about 1600 confirmed locally acquired human cases, 39% of which were in Italy [6]. Since then, Italy has been one of the most highly affected countries in Europe [7], with up to 723 human notified cases reported in 2022—some of which were fatal—mainly in its northern regions [8,9].

Control interventions against mosquito larvae should be the primary measures taken to reduce the adult abundance and risk of arbovirus outbreaks in Europe. Following the European Centre for Disease Control guidelines [10,11], the Italian Ministry of Health recommends preventing high mosquito densities and nuisance by targeting the larval developmental stage through an integrated approach that involves the removal of larval breeding sites or, when this is not feasible, the use of larvicide products. Pyrethroid spraying is recommended only in cases of a very high nuisance or autochthonous arbovirus circulation [12]. Under the current Biocidal Products Regulation (BPR, Regulation (EU) 528/2012 [13]), authorized larvicides in Europe are (i) microbial bacterial toxins *Bacillus thuringiensis* var. *israelensis* (Bti) and *Bacillus* (*Lysinibacillus*) *sphaericus* (Bs), extensively used against mosquito larvae, although their efficiency may vary under different ecological conditions [14,15]; (ii) diflubenzuron (DFB), a chitin synthesis inhibitor that interrupts the normal development of larvae into adults by interacting with chitin synthase 1 (*chs-1*), the enzyme responsible for chitin synthesis in the cuticle [16], and one of the most widely utilized mosquito larvicides in Europe [17]; and (iii) hormonal insect growth regulators (e.g., methoprene and pyriproxyfen), which have been used to a lesser extent. Temephos, a neurotoxic organophosphate widely used worldwide for several decades, is no longer authorized for mosquito control in Europe.

A significant issue arising from the limited variety of available insecticides authorized against mosquitoes, coupled with their widespread use in mosquito and agricultural pest control, is the development and spread of insecticide resistance [18]. Diflubenzuron resistance in mosquitoes was detected for the first time in 2015 in *Cx. pipiens* populations sampled from Emilia-Romagna (Italy), at high levels with a 128-fold resistance ratio (RR) [19]. The DFB resistance phenotype was associated with two mutations at the amino acid I1043 of the *chs-1* gene, resulting in the substitution of isoleucine with leucine (hereafter I1043L) or methionine (hereafter I1043M), targeting the DFB binding site. A third mutation at the same position of the *chs-1* gene results in the substitution of isoleucine with phenylalanine (hereafter I1043F), as was later identified by Fotakis et al. (2020) [20]. These mutations were introduced and functionally characterized in the *Drosophila melanogaster chs-1* gene using the CRISPR/Cas9 genome-editing method, demonstrating that they confer significant resistance, although at different levels: the I to F mutation results in an RR >15,000-fold; the I to M mutation results in an RR ranging from 2900 to 15,000-fold; and the I to L mutation in an RR > 20-fold [16,19]. Lucchesi et al. (2022) [21] detected in a resistant 1043M *Cx. pipiens* colony a constitutive 11-fold over-expression of the *chs-1* gene and an increase in cuticle thickness and cuticular chitin content, possibly contributing to resistance, as well as to the intensity of the resistant phenotype. Phylogenetic analyses showed independent origins of the I1043M, I1043L and I1043F mutations from different susceptible alleles, as well as multiple independent origins of the I1043M and I1043F alleles from different countries (Italy and Turkey) [22].

The widespread presence of I1043L and I1043M mutations was recorded in *Cx. pipiens* populations from the Emilia-Romagna region, with a clear pattern of increasing resistant allele frequencies moving from the western towards the eastern provinces, with allelic frequencies up to 77% at coastal sites [23]. The high frequencies of mutations observed in *Cx. pipiens* populations in the eastern provinces have been attributed to selection pressures due initially to intensive agricultural DFB applications against orchard pests during the 1980s and 1990s, followed by the adoption of DFB for mosquito control starting in the 2000s, when resistant mutations spread from rural to urban areas, indicating that agricultural selection was further boosted by public health applications [24].

The presence, frequency, and geographic distribution of DFB resistance mutations in *Cx. pipiens* beyond the Emilia-Romagna region in Italy have been investigated in France [20,23], Greece [20,23,25], Israel [20], Portugal [20] Turkey [22,26], and Armenia [27]. I1043L/M/F mutations have been found only in France (I1043F = 4.2%; [20]), Greece (I1043F = 8%; [25]), and Turkey (I1043L = 15.7–37.5% and I1043M = 25–52.7%; [22,26]).

The aim of the present study was to assess the circulation of 1043 resistance alleles in *Cx. pipiens* populations across Italy outside Emilia-Romagna, with a focus on the Veneto region.

## 2. Materials and Methods

### 2.1. Field Sampling Collections

**Figure 1:**
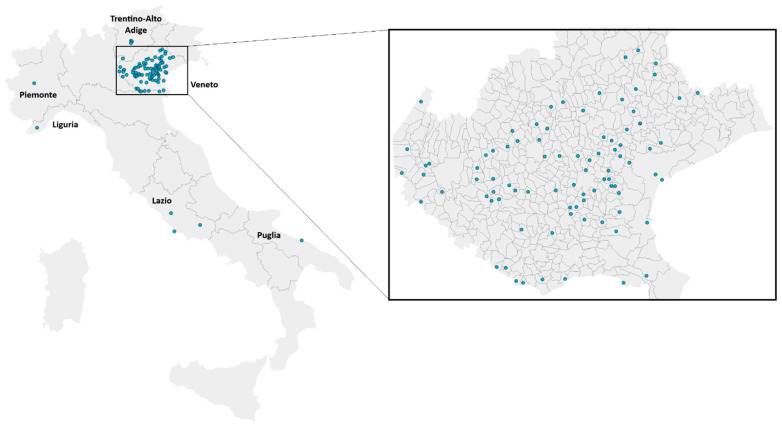
Mapping of the 96 sampling sites (blue dots) in 6 Italian regions (Trentino-Alto Adige, Veneto, Piemonte, Liguria, Lazio, and Puglia) (**left**). Detail of the 87 sites (blue dots) in the Veneto region (**right**).

*Culex pipiens* specimens were collected as egg rafts or by larval dipping Table S1 between 2016 and 2023 at several breeding sites (es. catch basins, fountains, ditches) from 96 sampling sites in 6 Italian regions (Trentino-Alto Adige, Veneto, Piemonte, Liguria, Lazio, and Puglia), with a particular focus on the Veneto region (87 sites, Figure 1, Table A1), where sampling was carried out as part of a mosquito monitoring program implemented by the Veneto region in collaboration with authorized technicians (Entostudio srl, Ponte San Nicolò, Italy). As part of this program, most samples were collected from catch basins during routine inspections for quality control of larvicide treatments applied by pest control companies. At each sampling site, larval samples were collected from at least five breeding sites to reduce the probability of collecting isofemale mosquitoes.

Mosquito larvae or emerged adults were identified morphologically to the species level [3,28] and stored in silica gel (adults) or 70% ethanol (adults and larvae). Sampled egg rafts were allowed to hatch under insectary conditions and larvae reared to the adult stage before being stored in silica gel or ethanol. When specimens from more than one year were available, they were pooled and overall frequencies were computed.

### 2.2. Genotyping of Diflubenzuron Resistance Mutations

Genomic DNA was extracted from the legs of single *Cx. pipiens* adults, or from field collected larvae using the DNAzol method following the Rider et al. (2012) [29] protocol, and dissolved in 30 μL or 50 μL of double-distilled water, respectively.

As described by Grigoraki et al. (2017) [19], the I1043L mutation was genotyped by allele-specific PCR (AS-PCR), while the I1043M mutation was genotyped by a PCR-RFLP diagnostic assay using the Hin1II restriction endonuclease.

For a subset of 460 specimens, sequencing of the 352 bp amplicons obtained using the External_F and External_R primers [19] was performed; this allowed us to confirm the genotyping results and to investigate the presence of the allele 1043F, for which no PCR-approach is yet available [20]. The obtained amplicons were sent to Eurofins Genomics Europe (Germany) for purification and sequencing which was performed using the Sanger method [30].

## 3. Results

A total of 1032 *Cx. pipiens* collected in Italy from 96 sites (3 in Trentino-Alto Adige, 87 in Veneto, 1 in Piemonte, 1 in Liguria, 3 in Lazio, and 1 in Puglia; Figure 1) were genotyped by AS-PCR and PCR-RFLP for the detection of the I1043L/M DFB-resistant mutations, respectively (Table 1; Table A1). Sequencing of 460 specimens allowed us to confirm the genotyping results, while no specimen carrying 1043F allele was detected.

**Figure 2:**
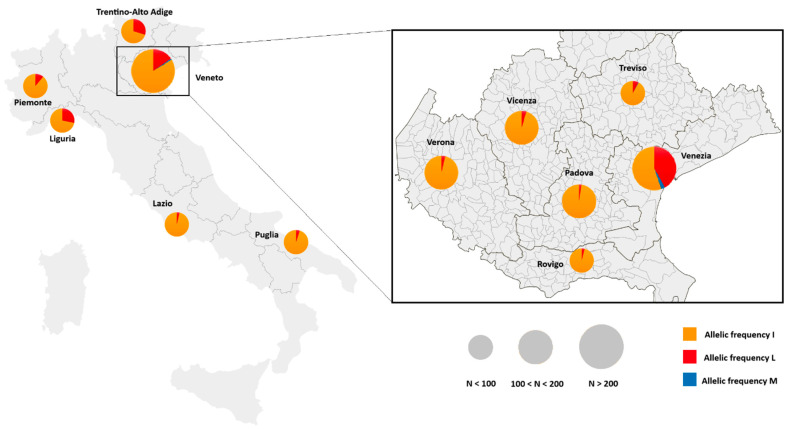
Map of average allelic frequencies of I1043L/M mutations associated with resistance to diflubenzuron in *Culex pipiens* samples from six Italian regions (**left**) and from six provinces in the Veneto region (**right**). Orange = wild-type I1043 allele; red = mutated 1043L allele; blue = mutated 1043M allele. Pie sizes are proportional to the sampling size, as indicated in the figure.

Allele 1043L was detected in all analyzed regions, with frequencies per site (considering only sites with ≥10 specimens genotyped) ranging between 19% and 36% in Trentino-Alto Adige, 0–91% in Veneto, 11% in Piemonte, 28% in Liguria, 0–8% in Lazio, and 5% in Puglia (Figure 2). The I1043M mutation was detected only at three sites of the Veneto region at frequencies ≤11%.

In the Veneto region, the mutated 1043L allele was detected in all six provinces analyzed (Figure 2), with frequencies ranging from 2% (Padova) to 42% (Venezia). Overall, the allele was detected in 26 out of 87 municipalities, with frequencies per site varying from 2% to 91% (considering only 10/26 sites with at least 10 specimens genotyped). The highest frequencies per site were detected at two sites in the Venetian lagoon—i.e., Venezia (80%) and Lido di Venezia (91%)—where 1043M allele was also detected, at frequencies of 6% (Lido di Venezia) and 11% (Chioggia) (Table A1). Furthermore, 1043M allele was detected at one site in Treviso province (San Vendemiano: 6%) (Table A1).

Homozygotes for 1043L allele were detected in Trentino-Alto Adige (Trento province: 14%), Liguria (Imperia: 19%), Veneto (at 19 out the 87 sites across all the six provinces, at frequencies ranging from 2 to 35% per province), and Lazio (Frosinone: 5%). Sites with the highest frequencies of 1043L homozygotes—at sites with at least 10 genotyped specimens—were detected again in Lido di Venezia (85%) and Venezia (80%). Homozygotes for 1043M allele were detected only at one site, i.e., Chioggia in the Venetian lagoon, at a frequency of 9%. Specimens carrying both mutated alleles (1043L/1043M) in heterozygosis were detected at two sites in Veneto (San Vendemiano, Treviso province: 1%, Lido di Venezia, Venezia province: 4%).

## 4. Discussion

Assessing the spread of resistance to DFB by performing bioassays on live larvae is challenging and time consuming due to the DFB mechanism of action, which does not induce a rapid killing effect, but prevents larvae/pupae from moulting. PCR-based diagnostics for monitoring I1043L/M mutations at amino acid 1043 of the *chs-1* gene in *Cx. pipiens* have been available since 2017—following strong evidence of their association with DFB-resistant phenotypes—and represent a valid and cost-effective alternative to bioassays for monitoring the insurgence and spread of DFB resistance in field populations [19]. These approaches have mostly been exploited to genotype *Cx. pipiens* populations in the River Po plain in the Emilia-Romagna region in northeast Italy, highlighting the widespread presence of I1043L and I1043M resistant mutations and the highest frequencies in the eastern, coastal areas.

The present study provides evidence of a widespread presence of I1043L mutation across Italy, the presence of mutation I1043M—associated with the strongest resistance to DFB [16,19]—only at three sites in Veneto, and an absence of allele 1043F in the analyzed samples.

Allele 1043F, detected previously in the Emilia-Romagna region, seems to be associated with the strongest resistance phenotype [20,22]. Unfortunately, this allele goes undetected with the available PCR approaches, meaning sequencing is necessary to reveal its presence. Since it was not possible to sequence all the analyzed specimens, we cannot exclude completelythe presence of this allele, at least at a low frequency, among the analyzed populations. A further major limit of this study is the reduced number of samples analyzed from regions other than Veneto; the results obtained have thus to be taken as a preliminary record pointing to the need to increase the monitoring of these mutations in Italy.

On the other hand, the dataset is more extensive for the Veneto region, where samples were collected from 87 municipalities in the framework of a regional mosquito control monitoring program, which performs routine larval collections from catch basins for quality control of larvicide treatments largely based on DFB [31]. Despite the sampling scheme resulting in relatively small sample sizes per single site, by analyzing the results from different sites within a province, a clear pattern was revealed. In inland provinces (i.e., Padova, Treviso, Verona, Vicenza), the 1043L allele was observed in only 4 out of 22 sites with ≥10 individuals genotyped, at frequencies ranging between 5% and 20%. However, in the coastal province of Venezia, 1043L allele was found in 6 out of 10 municipalities where ≥10 specimens were genotyped, and at extremely high frequencies in samples from the Venetian lagoon over five years (Lido di Venezia: 97%, 74%, and 95% in 2019, 2020, and 2023, respectively, Table A2; 80% in Venezia in 2022). Moreover, the 1043M allele was also observed in the Venetian lagoon, at frequencies of 6% in Lido di Venezia and 11% in Chioggia.

**Figure 3:**
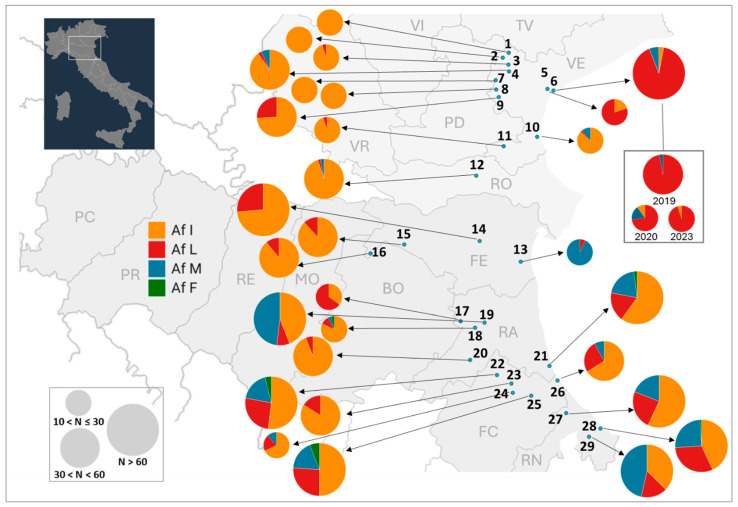
Map of allelic frequencies of I1043 mutations associated with resistance to diflubenzuron in *Culex pipiens* samples from 29 sites (blue dots) in coastal provinces sampled from 2016 to 2023. The dataset includes data obtained in the present work, as well as literature data (Table A3). When a single site was sampled more than once, data were pooled to provide an overall picture of the allele frequencies, not considering variation among years. Orange = wild-type I1043 allele; red = mutated 1043L allele; blue = mutated 1043M allele. Provinces in Veneto: VE = Venezia, RO = Rovigo. Provinces in Emilia-Romagna: FE = Ferrara; RA = Ravenna; FC = Forlì-Cesena; RI = Rimini. Pie sizes are proportional to the sampling size, as indicated in the figure. Af = allelic frequency.

Focusing on coastal provinces and merging the present data with previous datafrom other sites in the Veneto and Emilia-Romagna regions (Table A3) revealed consistent results, with the highest frequencies of resistant alleles at the easternmost sites (Figure 3), although the 1043M allele appeared generally more frequently than in Veneto. However, the frequencies of the 1043L allele > 90% observed in Lido di Venezia largely exceed the frequencies reported from Emilia-Romagna, and they are the highest ever reported in field *Cx. pipiens* populations.

The emerging scenario reveals hot spots of highly resistant populations at sites along the Adriatic coast characterized by extensive seasonal insecticide treatments against mosquitoes due to the sites’ high touristic use. Interestingly, *Cx. pipiens* populations from coastal Adriatic sites are also found to be highly resistant to pyrethroids due to the implementation of calendar-based ground pyrethroid spraying in parallel to larvicide treatments in rain catch basins [32], and frequencies of resistant alleles are also generally higher in *Aedes albopictus* from coastal rather than inland sites [33]. Notably, the largest outbreaks of Chikungunya (2007) [34] and Dengue (2024) [35] in Italy have occurred at coastal tourist sites on the Adriatic coast. It is not possible to discriminate at this stage whether the outbreaks were triggered by a higher mosquito abundance due to strong resistance to insecticide in the local populations, or whether increased insecticide resistance is to be ascribed to the greater use of insecticides during and after the outbreaks. Porretta et al. (2019; 2022) [23,24] hypothesized that the 2007 Chikungunya outbreak boosted the selection of DFB resistance not only in the target species, *Ae. albopictus*, but also in sympatric *Cx. pipiens* populations, following extensive DFB use in the year after the outbreak (as already hypothesized for pyrethroid target-site resistance after the Chikungunya outbreak in the Lazio region; [36]).

Overall, lower frequencies of resistant alleles were observed in most *Cx. pipiens* populations from inland provinces of Veneto, as already shown for Emilia-Romagna [19,20,23,24], suggesting lower selective pressures despite the regular implementation in both regions of larvicide treatments (mostly by DFB or *Bacillus*) over the whole summer season, as has been the case for several years. In Emilia-Romagna, differences in resistant allele frequencies between inland areas and the coast have been explained mainly by increased selective pressure on the coast due to intensive treatments performed to reduce the nuisance to tourists and for agricultural purposes [23]. Analyzed populations from Veneto were all collected in urban/periurban areas with similar public mosquito control programs and thus similar selective pressure, but likely maintain gene flow with rural populations with less extensive/homogeneous DFB use. On the other hand, the sites in the Venetian lagoon are on highly touristic islands, and this may imply more intensive mosquito control activities by private subjects (e.g., hotels, resorts) in addition to public treatments. Notably, in these locations, the abundance of breeding sites different from rain catch basins may be lower than inland. Moreover, the sites are connected to the mainland only by bridges (Venezia and Chioggia) or by boat (Lido di Venezia) and thus the local mosquito populations are more isolated, meaning gene flow with other populations from areas with lower insecticide pressure (and thus a lower resistance allele frequency) is restricted.

It is interesting to note that data reported in Figure 3 refer to samples collected between 2016 and 2023. Porretta et al. (2022) [24] did not find any resistant alleles in samples collected on the Adriatic coast in Emilia-Romagna between 1987 and 1993, although they found the 1043M allele at frequencies up to 26% in samples collected in Padova province (Veneto) in 1986–87, from where we detected only extremely low frequencies of the 1043L allele in 2020–2022. Despite the likely bias due to the small sizes of both samples, this preliminary evidence raises questions about the selection dynamics of these resistant alleles and their fitness costs. Such studies are highly necessary to understand the spread of resistance and to implement resistance management programs. Strong fitness costs, as, for example, are suggested for DFB-resistant *Cx. pipiens* form *molestus* [37], which has a highly reduced winter survival, could contribute to maintaining resistance limited to some focal sites with high insecticidal pressure, as observed within the present study. Also, detailed information on insecticide usage by public and private entities is necessary to allow a better interpretation of the data and enhance our understanding of selective processes that are ongoing, but such information is unfortunately not available to date.

These questions could only be answered by a longitudinal study, which requires large samples collected during the last 20 years from sites where insecticide pressure over the years can be precisely assessed. Regrettably, such samples are not available to the authors of this paper.

## 5. Conclusions

The overall picture emerging from this and the other few studies focusing on resistance to DFB in European populations of *Cx. pipiens* is a widespread presence of I1043L/M mutations in Italy. In Veneto territory, the frequencies of mutations are mostly low, despite most municipalities regularly implementing seasonal larvicide treatments in catch basins, and despite DFB being the most common, although not the only, larvicide used [38]. Samples were mostly collected in treated (mainly with DFB, but also with other larvicides) catch basins sampled in the framework of routine inspections for quality control of longstanding seasonal larvicide treatments applied by pest control companies at urban/periurban sites. Our data suggest that, despite DFB having been used routinely since 2000 for mosquito control in Italy [24], this seems to be not sufficient to select high frequencies of 1043 resistant alleles in urban and periurban mosquito populations. The low DFB resistance allele frequencies reported from inland Emilia-Romagna [19,20,23,24], as well as France and Greece [20,25] suggest a similar situation in Europe. More detailed knowledge of the local larvicide interventions is needed to confirm this hypothesis, but is very difficult to obtain in a non-endemic area such Italy, where treatments are mostly performed by private citizens to reduce the mosquito nuisance. The available evidence encourages novel studies to understand the potential fitness cost of these alleles.

On the other hand, the results also suggest that under specific conditions such as those observed in the Venetian lagoon, the resistant alleles reach frequencies close to fixation, which implies that DFB treatment may be ineffective.

It is relevant to note that DFB will not be allowed for mosquito larval control in Italy after June 2025, due to its toxicity for aquatic life. However, this will likely not completely overcome the risk of ineffective treatments by other larvicides in populations with very high 1043 mutations. In fact, the I1043M mutation has been shown to affect the efficacy of DFB molecules by causing not only an aminoacidic change at the DFB target site but also over-expression of the *chs-1* gene, which positively affects cuticle deposition [21]. Since an enhanced cuticle thickness has been documented to reduce the penetration of several insecticide molecules [39], this implies that mutations at the *chs-1* site may broaden the resistance of *Cx. pipiens* to other larvicides, as well as to pyrethroids. It is thus relevant to test the resistance of field populations showing high frequencies of 1043 mutations to other insecticides.

More in general, the results obtained highlight that genotyping of mutations associated with insecticide resistance allows us to identify areas—such as the Venetian lagoon or the Romagna Riviera—where the selective pressure exerted by insecticide use is high as to select high frequencies of resistant genotypes. Despite the WHO recommending that resistance allele monitoring is to be prioritized at the sites with the highest insecticidal pressure, these sites are often difficult to identify given the usage of insecticides not only for mosquito control by public institutions but also in agriculture and by private subjects. Specifically, the results on 1043 mutation genotyping so far suggest not only that resistance monitoring should be prioritized at coastal tourist sites but also that at these sites, larvicidal active principles should be rotated to prevent the establishment of high-resistance populations. Indeed, while pyrethroids are the only active principle authorized in Europe for adult mosquito control, limiting the possibility of rotation between products with different modes of action, the availability of different larvicides actually allows for rotations of different products. Optimal planning of control interventions, including rotation of products, will in any case benefit from a previous evaluation of their effectiveness and the possible presence of resistance. The option to rotate products can be crucial for reducing the frequencies of circulating resistant alleles.

## Figures and Tables

**Table 1:** Average genotypic and allelic frequencies of I1043 mutations associated with resistance to diflubenzuron in *Culex pipiens* samples from 12 provinces across Italy. Date = sampling years. N° sites = number of sampled sites. N° samples = number of *Cx. pipiens* individuals analyzed. II = homozygous I1043I wild-type specimens. IL = heterozygous I1043I/I1043L specimens. IM = heterozygous I1043I/I1043M specimens. LL = homozygous I1043L mutated specimens. LM = heterozygous I1043L/I1043M specimens. MM = homozygous I1043M mutated specimens. Gf = genotypic frequency. Af = allelic frequency.

Region-Province	Date	N° Sites	N° Samples	Genotypic Frequencies						Allelic Freq		
				Gf II	Gf IL	Gf IM	Gf LL	Gf LM	Gf MM	Af L	Af M	Af I
Trentino-Alto Adige-Trento	2017–2020	3	84	0.55	0.31	0	0.14	0	0	0.30	0	0.70
Veneto-Padova	2020–2022	19	181	0.97	0.01	0	0.02	0	0	0.02	0	0.98
Veneto-Rovigo	2020–2022	8	76	0.96	0	0	0.04	0	0	0.04	0	0.96
Veneto-Treviso	2020–2022	14	86	0.91	0.02	0	0.06	0.01	0	0.08	0.01	0.92
Veneto-Venezia	2019–2023	16	252	0.50	0.09	0.004	0.35	0.04	0.01	0.42	0.03	0.55
Veneto-Verona	2020–2022	16	120	0.95	0.03	0	0.03	0	0	0.04	0	0.96
Veneto-Vicenza	2020–2022	14	124	0.94	0.02	0	0.03	0	0	0.04	0	0.96
Piemonte-Torino	2017	1	18	0.78	0.22	0	0	0	0	0.11	0	0.89
Liguria-Imperia	2018	1	16	0.63	0.19	0	0.19	0	0	0.28	0	0.72
Lazio-Frosinone	2017	1	19	0.89	0.05	0	0.05	0	0	0.08	0	0.92
Lazio-Roma	2016–2017	2	36	0.97	0.03	0	0	0	0	0.01	0	0.99
Puglia-Bari	2017–2018	1	20	0.90	0.10	0	0	0	0	0.05	0	0.95
**Total**	**2016–2023**	**96**	**1032**	**0.80**	**0.07**	**0**	**0.12**	**0.01**	**0**	**0.16**	**0.01**	**0.83**

## Data Availability

All data are available within the article and in Appendix A.

## Supplementary Materials

The following supporting information can be downloaded at: https://www.mdpi.com/article/10.3390/insects16020204/s1, Table S1: Details on sampling methods and genotyping results per sampling site/year and province.

## Appendix A

**Table A1:** Average genotypic and allelic frequencies of I1043 mutations associated with resistance to diflubenzuron in *Culex pipiens* samples from 96 municipalities across Italy. Specimens collected in more than one year were pooled and overall frequencies were computed. Date = sampling years. N° sites = number of sampled sites. N° samples = number of *Cx. pipiens* individuals analyzed. II = homozygous I1043I wild-type specimen. IL = heterozygous I1043I/I1043L specimen. IM = heterozygous I1043I/I1043M specimen. LL = homozygous I1043L mutated specimen. LM = heterozygous I1043L/I1043M specimen. MM = homozygous I1043M mutated specimen. Gf = genotypic frequency. Af = allelic frequency.

Site	Date	N	Genotypic Frequencies						Allelic Freq		
			Gf II	Gf IL	Gf IM	Gf LL	Gf LM	Gf MM	Af L	Af M	Af I
Mezzolombardo	2020	48	0.44	0.40	0	0.17	0	0	0.36	0	0.64
San Michele All’adige	2017	16	0.69	0.25	0	0.06	0	0	0.19	0	0.81
Zambana	2017	20	0.70	0.15	0	0.15	0	0	0.23	0	0.78
Trento Province	2017–2020	84	0.55	0.31	0	0.14	0	0	0.30	0	0.70
Albignasego	2022	10	0.90	0	0	0.10	0	0	0.10	0	0.90
Battaglia Terme	2022	2	1.00	0	0	0	0	0	0	0	1.00
Borgoricco	2022	5	1.00	0	0	0	0	0	0	0	1.00
Campodarsego	2021–2022	8	1.00	0	0	0	0	0	0	0	1.00
Candiana	2022	6	1.00	0	0	0	0	0	0	0	1.00
Casale di Scodosia	2022	5	1.00	0	0	0	0	0	0	0	1.00
Codevigo	2022	8	0.88	0.13	0	0	0	0	0.06	0	0.94
Conselve	2020	6	1.00	0	0	0	0	0	0	0	1.00
Curtarolo	2020–2022	19	0.84	0.05	0	0.11	0	0	0.13	0	0.87
Due Carrare	2022	8	1.00	0	0	0	0	0	0	0	1.00
Maserà di Padova	2022	10	1.00	0	0	0	0	0	0	0	1.00
Padova	2022	9	1.00	0	0	0	0	0	0	0	1.00
Pernumia	2021	21	1.00	0	0	0	0	0	0	0	1.00
Piombino Dese	2021	11	1.00	0	0	0	0	0	0	0	1.00
Ponte San Nicolò	2020	4	1.00	0	0	0	0	0	0	0	1.00
Teolo	2020–2022	13	1.00	0	0	0	0	0	0	0	1.00
Trebaseleghe	2020–2022	26	1.00	0	0	0	0	0	0	0	1.00
Vigodarzere	2022	3	1.00	0	0	0	0	0	0	0	1.00
Villa Estense	2020	7	1.00	0	0	0	0	0	0	0	1.00
Padova Province	2020–2022	181	0.97	0.01	0	0.02	0	0	0.02	0	0.98
Ariano nel Polesine	2022	10	1.00	0	0	0	0	0	0	0	1.00
Castelmassa	2022	9	1.00	0	0	0	0	0	0	0	1.00
Ceneselli	2022	9	0.89	0	0	0.11	0	0	0.11	0	0.89
Ficarolo	2022	9	0.89	0	0	0.11	0	0	0.11	0	0.89
Fiesso Umbertiano	2022	10	1.00	0	0	0	0	0	0	0	1.00
Gaiba	2022	10	1.00	0	0	0	0	0	0	0	1.00
Polesella	2020	9	0.89	0	0	0.11	0	0	0.11	0	0.89
Rovigo	2022	10	1.00	0	0	0	0	0	0	0	1.00
Rovigo Province	2020–2022	76	0.96	0	0	0.04	0	0	0.04	0	0.96
Cison di Valmarino	2020	1	0	0	0	1.00	0	0	1.00	0	0
Colle Umberto	2021	6	1.00	0	0	0	0	0	0	0	1.00
Gorgo al Monticano	2022	1	1.00	0	0	0	0	0	0	0	1.00
Loria	2020	4	1.00	0	0	0	0	0	0	0	1.00
Maser	2022	8	1.00	0	0	0	0	0	0	0	1.00
Meduna di Livenza	2021–2022	19	1.00	0	0	0	0	0	0	0	1.00
Nervesa della Battaglia	2020	3	1.00	0	0	0	0	0	0	0	1.00
Oderzo	2022	10	1.00	0	0	0	0	0	0	0	1.00
Pederobba	2020	2	0.50	0.50	0	0	0	0	0.25	0	0.75
Ponzano Veneto	2022	5	1.00	0	0	0	0	0	0	0	1.00
Quinto di Treviso	2020	2	0	0	0	1.00	0	0	1.00	0	0
Revine Lago	2021	15	1.00	0	0	0	0	0	0	0	1.00
San Vendemiano	2020	8	0.63	0.13	0	0.13	0.13	0	0.25	0.06	0.69
Volpago del Montello	2020	2	0.50	0	0	0.50	0	0	0.50	0	0.50
Treviso Province	2020–2022	86	0.91	0.02	0	0.06	0.01	0	0.08	0.01	0.92
Campagna Lupia	2022	5	1.00	0	0	0	0	0	0	0	1.00
Camponogara	2022	5	1.00	0	0	0	0	0	0	0	1.00
Chioggia	2020–2022	23	0.83	0.04	0.04	0	0	0.09	0.02	0.11	0.87
Cona	2020–2022	11	0.91	0.09	0	0	0	0	0.05	0	0.95
Fiesso d’Artico	2022	10	1.00	0.0	0	0	0	0	0	0	1.00
Fossò	2019–2020	42	0.57	0.33	0	0.10	0	0	0.26	0	0.74
Lido di Venezia	2019–2023	86	0.02	0.01	0	0.85	0.12	0	0.91	0.06	0.03
Marcon	2020	6	0.67	0	0	0.33	0	0	0.33	0	0.67
Noale	2022	10	1.00	0	0	0	0	0	0	0	1.00
Pianiga	2022	10	1.00	0	0	0	0	0	0	0	1.00
Quarto d’Altino	2020	6	0	0.83	0	0.17	0	0	0.58	0	0.42
Salzano	2022	10	0.90	0.10	0	0	0	0	0.05	0	0.95
Scorzè	2022	10	1.00	0	0	0	0	0	0	0	1.00
Spinea	2020	5	0.80	0	0	0.20	0	0	0.20	0	0.80
Stra	2021	3	1.00	0	0	0	0	0	0	0	1.00
Venezia	2022	10	0.20	0	0	0.80	0	0	0.80	0	0.20
Venezia Province	2019–2023	252	0.50	0.09	0.004	0.35	0.04	0.01	0.42	0.03	0.55
Albaredo d’Adige	2020	5	0.20	0.40	0	0.40	0	0	0.60	0	0.40
Arcole	2022	9	1.00	0	0	0	0	0	0	0	1.00
Bardolino	2022	7	1.00	0	0	0	0	0	0	0	1.00
Bussolengo	2022	6	1.00	0	0	0	0	0	0	0	1.00
Caldiero	2021	1	1.00	0	0	0	0	0	0	0	1.00
Castel d’Azzano	2022	10	1.00	0	0	0	0	0	0	0	1.00
Illasi	2021	2	1.00	0	0	0	0	0	0	0	1.00
Malcesine	2022	10	1.00	0	0	0	0	0	0	0	1.00
Monteforte d’Alpone	2020	4	0.75	0.25	0	0	0	0	0.13	0	0.88
Mozzecane	2020–2022	18	1.00	0	0	0	0	0	0	0	1.00
Pescantina	2020	9	0.89	0	0	0.11	0	0	0.11	0	0.89
Peschiera del Garda	2022	8	1.00	0	0	0	0	0	0	0	1.00
Ronco All’Adige	2022	10	1.00	0	0	0	0	0	0	0	1.00
San Giovanni Ilarione	2022	10	1.00	0	0	0	0	0	0	0	1.00
Sona	2020	1	1.00	0	0	0	0	0	0	0	1.00
Veronella	2022	10	1.00	0	0	0	0	0	0	0	1.00
Verona Province	2020–2022	120	0.95	0.03	0	0.03	0	0	0.04	0	0.96
Alonte	2020–2021	6	1.00	0	0	0	0	0	0	0	1.00
Bassano del Grappa	2022	10	1.00	0	0	0	0	0	0	0	1.00
Bressanvido	2022	10	1.00	0	0	0	0	0	0	0	1.00
Camisano Vicentino	2022	4	1.00	0	0	0	0	0	0	0	1.00
Chiampo	2022	10	0.90	0.10	0	0	0	0	0.05	0	0.95
Gambugliano	2021	3	1.00	0	0	0	0	0	0	0	1.00
Lonigo	2020	7	1.00	0	0	0	0	0	0	0	1.00
Marostica	2020	7	1.00	0	0	0	0	0	0	0	1.00
Montecchio Precalcino	2020	2	0	0	0	1.00	0	0	1.00	0	0
Monticello Conte Otto	2020–2021	15	1.00	0	0	0	0	0	0	0	1.00
Sossano	2021	6	1.00	0	0	0	0	0	0	0	1.00
Torri di Quartesolo	2021	7	1.00	0	0	0	0	0	0	0	1.00
Trissino	2020–2022	15	0.73	0.13	0	0.13	0	0	0.20	0	0.80
Vicenza	2021–2022	22	1.00	0	0	0	0	0	0	0	1.00
Vicenza Province	2020–2022	124	0.77	0.02	0	0.03	0	0	0.04	0	0.78
Torino	2017	18	0.78	0.22	0	0	0	0	0.11	0	0.89
Torino Province	2017	18	0.78	0.22	0	0	0	0	0.11	0	0.89
Imperia	2018	16	0.63	0.19	0	0.19	0	0	0.28	0	0.72
Imperia Province	2018	16	0.63	0.19	0	0.19	0	0	0.28	0	0.72
Anzio	2017	18	1.00	0	0	0	0	0	0	0	1.00
Roma	2016	18	0.94	0.06	0	0	0	0	0.03	0	0.97
Roma Province	2016–2017	36	0.97	0.03	0	0	0	0	0.01	0	0.99
Strangolagalli	2017	19	0.89	0.05	0	0.05	0	0	0.08	0	0.92
Frosinone Province	2017	19	0.89	0.05	0	0.05	0	0	0.08	0	0.92
Bari	2017–2018	20	0.90	0.10	0	0	0	0	0.05	0	0.95
Bari Province	2017–2018	20	0.90	0.10	0	0	0	0	0.05	0	0.95

**Table A2:** Average genotypic and allelic frequencies of I1043 mutations associated with resistance to diflubenzuron in *Culex pipiens* samples collected from Lido di Venezia in different years. Date = sampling years. N° sites = number of sampled sites. N° samples = number of *Cx. pipiens* individuals analyzed. II = homozygous I1043I wild-type specimen. IL = heterozygous I1043I/I1043L specimen. IM = heterozygous I1043I/I1043M specimen. LL = homozygous I1043L mutated specimen. LM = heterozygous I1043L/I1043M specimen. MM = homozygous I1043M mutated specimen. Gf = genotypic frequency. Af = allelic frequency.

Region	Province	Site	Date	N	Gf II	Gf IL	Gf IM	Gf LL	Gf LM	Gf MM	Af L	Af M	Af I
Veneto	Venezia	Lido di Venezia	2019	55	0	0	0	0.95	0.05	0	0.97	0.03	0
Veneto	Venezia	Lido di Venezia	2020	21	0.10	0	0	0.57	0.33	0	0.74	0.17	0.10
Veneto	Venezia	Lido di Venezia	2023	10	0	0.10	0	0.90	0	0	0.95	0	0.05

**Table A3:** Average allelic frequencies of I1043L/M/F mutations associated with resistance to diflubenzuron in *Culex pipiens* samples from 47 municipalities in the Veneto and Emilia-Romagna regions from papers published so far. Cod = codes corresponding to the numbers on the map (Figure 3). Date = sampling years. N = number of *Cx. pipiens* individuals analyzed. Af = allelic frequency. I = I1043 susceptible allele; L = 1043L mutant allele; M = 1043M mutant allele; F = 1043F mutant allele. Paper = source papers of the analyzed samples [19,20,23,24].

Cod	Region	Province	Municipality	Date	N	Af I	Af L	Af M	Af F	Paper
1	Veneto	Venezia	Scorzè	2022	10	1.00	0	0	0	Present paper
2	Veneto	Venezia	Noale	2022	10	1.00	0	0	0	Present paper
3	Veneto	Venezia	Salzano	2022	10	0.95	0.05	0	0	Present paper
4	Veneto	Venezia	Mirano	2018	33	0.90	0.03	0.07	0	Porretta 2022
5	Veneto	Venezia	Venezia	2022	10	0.20	0.80	0	0	Present paper
6	Veneto	Venezia	Lido di Venezia	2019–2023	86	0.03	0.91	0.06	0	Present paper
7	Veneto	Venezia	Pianiga	2022	10	1.00	0	0	0	Present paper
8	Veneto	Venezia	Fiesso d’Artico	2022	10	1.00	0	0	0	Present paper
9	Veneto	Venezia	Fossò	2019–2020	42	0.74	0.26	0	0	Present paper
10	Veneto	Venezia	Chioggia	2020–2022	23	0.87	0.02	0.11	0	Present paper
11	Veneto	Venezia	Cona	2020–2022	11	0.95	0.05	0	0	Present paper
12	Veneto	Rovigo	Rovigo	2018–2022	46	0.95	0.02	0.03	0	Porretta 2022; Present paper
13	Emilia-Romagna	Ferrara	Comacchio	2018	30	0	0.07	0.93	0	Fotakis 2020
14	Emilia-Romagna	Ferrara	Ferrara	2017–2020	87	0.73	0.26	0	0	Porretta 2019; Porretta 2022
15	Emilia-Romagna	Ferrara	Poggio Renatico	2017	47	0.88	0.12	0	0	Porretta 2019
16	Emilia-Romagna	Ferrara	Cento	2017	49	0.89	0.11	0	0	Porretta 2019
17	Emilia-Romagna	Ravenna	Massa Lombarda	2018	27	0.35	0.65	0	0	Fotakis 2020
18	Emilia-Romagna	Ravenna	Lugo	2018	30	0.82	0.10	0.05	0.03	Fotakis 2020
19	Emilia-Romagna	Ravenna	Ravenna	2016–2018	171	0.44	0.08	0.48	0	Grigoraki 2017; Porretta 2019; Porretta 2022
20	Emilia-Romagna	Ravenna	Faenza	2017	47	0.94	0.06	0	0	Porretta 2019
21	Emilia-Romagna	Ravenna	Cervia	2017	87	0.60	0.18	0.20	0.02	Porretta 2019; Fotakis 2020
22	Emilia-Romagna	Forlì-Cesena	Forlì	2017–2018	113	0.52	0.26	0.18	0.04	Porretta 2019; Fotakis 2020; Porretta 2022
23	Emilia-Romagna	Forlì-Cesena	Forlimpopoli	2017	48	0.84	0.16	0	0	Porretta 2019
24	Emilia-Romagna	Forlì-Cesena	Bertinoro	2018	30	0.68	0.20	0.12	0	Fotakis 2020
25	Emilia-Romagna	Forlì-Cesena	Cesena	2018–2020	65	0.50	0.26	0.18	0.06	Fotakis 2020; Porretta 2022
26	Emilia-Romagna	Forlì-Cesena	Cesenatico	2017	50	0.66	0.26	0.08	0	Porretta 2019
27	Emilia-Romagna	Rimini	Santarcangelo di Romagna	2017–2018	80	0.58	0.24	0.19	0	Porretta 2019; Fotakis 2020
28	Emilia-Romagna	Rimini	Riccione	2017–2018	77	0.43	0.31	0.25	0.01	Porretta 2019; Fotakis 2020
29	Emilia-Romagna	Rimini	Rimini	2017–2018	123	0.37	0.16	0.46	0	Porretta 2019; Fotakis 2020
